# *Neurobiology of Language*: Volume 5 Reviewers List

**DOI:** 10.1162/nol_x_00163

**Published:** 2024-12-03

Ayisha Al Busaidi

F.-Xavier Alario

Phillip Alday

Suhas Arehalli

Laura Batterink

Yannick Becker

Emmanuel Biau

Ferdinand Binkofski

Jason Bohland

Geert Booij

Ina Bornkessel-Schlesewsky

Mirjana Bozic

Jonathan Brennan

Giovanni Buccino

Talat Bulut

Marianne Casilio

Laurent Cohen

David Copland

David Corina

Brendan Costello

Jacqueline Cummine

Luc De Nil

Greig de Zubicaray

Andrew DeMarco

Dirk Den Ouden

Junhua Ding

Nai Ding

Julien Dirani

Nicolas Dumay

Karen Emmorey

Andreia Faria

John Gabrieli

Simone Gastaldon

Robin Gerrits

Laura Giglio

Blake Gimbel

Vincent Gracco

Margriet Groen

Eva Gutierrez-Sigut

Laura Gwilliams

Liberty Hamilton

Eleanor Elizabeth Hardinig

Olaf Hauk

Guillaume Herbet

Arild Hestvik

Paul Hoffman

Gina Humphreys

Maria Ivanova

Shailee Jain

Marc Joanisse

Efthymia Kapnoula

Itamar Kastner

Christian Kell

Ferenc Kemeny

Andrea Krott

Lynn Kurteff

Loïc Labache

Melinh K. Lai

Nicole Landi

Ellen Lau

Minna Lehtonen

Hanjun Liu

Steve Majerus

Emmanuel Mandonnet

Suhail Matar

William Matchin

David McAlpine

Philip J. Monahan

Maxime Montembeault

Angela Morgan

Alex Murphy

Elliot Murphy

Emily Beth Myers

Megan Nakamura

Nicole E. Neef

Anni Nora

Sebastian Ocklenburg

Yulia Oganian

Fulvia Palesi

Hyojin Park

Aurélie Pistono

Cristina Polito

Amy Ramage

Josef Rauschecker

Jana Reifegerste

Gail Robinson

Donovan Roediger

Ariel Rokem

Tatiana Schnur

Eleonore Smalle

Faye Smith

Brielle Stark

Melissa Dawn Stockbridge

Jakke Tamminen

Marco Tettamanti

Filiz Tezcan Semerci

Xing Tian

Sarah Tune

Tae Twomey

Michael Ullman

Maaike Vandermosten

Helena Verhelst

Gabriela Vigliocco

Yi Wei

Stephen M. Wilson

Maya Yablonski

Jason Yeatman

Caicai Zhang

Yang Zhang

Christina Zhao

JingJing Zhao

Vitor Zimmerer

Lauryn Zipse

